# Gender Differences in School Stress and Academic Satisfaction in Pre-Adolescents: The Role of Physical Activity

**DOI:** 10.3390/children12101282

**Published:** 2025-09-23

**Authors:** Josivaldo de Souza-Lima, Gerson Ferrari, Rodrigo Yáñez-Sepúlveda, Frano Giakoni-Ramírez, Catalina Muñoz-Strale, Javiera Alarcon-Aguilar, Maribel Parra-Saldias, Daniel Duclos-Bastias, Andrés Godoy-Cumillaf, Eugenio Merellano-Navarro, José Bruneau-Chávez, Claudio Farias-Valenzuela, Pedro Valdivia-Moral

**Affiliations:** 1 Facultad de Educación y Ciencias Sociales, Instituto del Deporte y Bienestar, Universidad Andres Bello, Las Condes, Santiago 7550000, Chile; rodrigo.yanez.s@unab.cl (R.Y.-S.); frano.giakoni@unab.cl (F.G.-R.); catalina.munoz@unab.cl (C.M.-S.); javiera.alarcon@unab.cl (J.A.-A.); 2 Facultad de Ciencias de la Educación, Universidad de Granada, 18071 Granada, Spain; pvaldivia@ugr.es; 3 Escuela de Ciencias de la Actividad Física, el Deporte y la Salud, Universidad de Santiago de Chile (USACH), Santiago 9170124, Chile; gerson.demoraes@usach.cl; 4 School of Medicine, Universidad Espíritu Santo, Samborondón 092301, Ecuador; 5 Departamento de Educación Física, Deporte y Recreación, Universidad de Atacama, Copiapó 1530000, Chile; maribel.parra@uda.cl; 6 GEO Research Group, Escuela de Educación Física, Pontificia Universidad Católica de Valparaíso, Valparaíso 2340021, Chile; daniel.duclos@pucv.cl; 7 METIS Research Lab, Facultad de Negocios y Tecnología, Universidad Alfonso X el Sabio (UAX), Avenida de la Universidad 1, 28691 Villanueva de la Cañada, Spain; 8 Grupo de Investigación en Educación Física, Salud y Calidad de Vida (EFISAL), Facultad de Educación, Universidad Autónoma de Chile, Temuco 4780000, Chile; andres.godoy@uautonoma.cl; 9 Department of Physical Activity Sciences, Faculty of Education Sciences, Universidad Católica del Maule, Talca 3530000, Chile; emerellano@ucm.cl; 10 Departamento de Educación Física, Deportes y Recreación, Universidad de la Frontera, Temuco 4811230, Chile; jose.bruneau@ufrontera.cl; 11 Escuela de Ciencias de la Actividad Física, Universidad de Las Américas, Santiago 7500975, Chile; claudio.farias.valenzuela@edu.udla.cl

**Keywords:** pre-adolescents, school stress, physical activity, gender differences, subjective well-being, boredom, academic satisfaction, multinational study

## Abstract

Background/Objectives: Gender differences in school stress and academic satisfaction among pre-adolescents remain underexplored, particularly in relation to physical activity as a potential protective factor. This study aimed to examine these differences and investigate the role of sports/exercise frequency in mitigating boredom and stress, controlling for school safety, using a large multinational dataset. Methods: Data from the International Survey of Children’s Well-Being (ISCWeB, third wave; N = 128,184 pre-adolescents aged 6–14 from 35 countries) were analyzed. Key variables included boredom and stress (0–10 scales), sports/exercise frequency (categorized as low: 0–2 days/week, medium: 3–4, high: 5–6), and school safety (0–4 scale). Descriptive statistics were stratified by gender and sports level. Multivariate analysis of variance (MANOVA) tested combined effects of sports level, gender, and their interaction, followed by univariate ANOVAs (Type II). Results: Modest gender differences were found in stress (boys: mean = 4.05; girls: mean = 4.16) and academic satisfaction (boys: 8.50; girls: 8.66), with similar distributions in physical activity variables. Higher sports frequency was associated with lower boredom (high: 4.00 vs. low: 4.46) and stress (high: 4.03 vs. low: 4.05). MANOVA confirmed a significant multivariate effect of sports level (Wilks’ lambda = 0.9984, F = 49.74, *p* < 0.0001), with marginal gender effect (*p* = 0.0525) and significant interaction (Wilks’ lambda = 0.9998, F = 6.59, *p* < 0.0001); effect sizes were small (partial η^2^ ≤ 0.014). School safety was a significant covariate (Wilks’ lambda = 0.9807, F = 1262.84, *p* < 0.0001, partial η^2^ ≈ 0.013). Conclusions: Physical activity modestly protects against school-related boredom and stress in pre-adolescents, with modest gender moderation. Findings support universal school programs promoting daily exercise, with gender-sensitive adaptations, to enhance emotional well-being and academic satisfaction.

## 1. Introduction

Subjective well-being (SWB) in children and pre-adolescents is a multifaceted construct that includes cognitive evaluations of life satisfaction and affective experiences of positive and negative emotions, serving as a pivotal indicator of overall psychological health and development [1,2]. Globally, SWB is linked to improved academic performance, social relationships, and long-term mental health outcomes, but it tends to decline as children approach adolescence due to increasing academic and social pressures. In school environments, where pre-adolescents spend most of their waking hours, factors such as peer interactions and academic demands significantly influence SWB, emphasizing the need to identify protective mechanisms that can sustain positive emotional states.

School-related stress is a prevalent issue among pre-adolescents, often stemming from high academic expectations, social conflicts, and perceived lack of safety, which can lead to emotional distress and reduced engagement [3,4]. Elevated stress levels are associated with lower academic satisfaction and increased risk of mental health problems, with meta-analyses reporting stress negatively impacts engagement and performance in youth [5]. Boredom, closely related to stress, further contributes to disengagement and motivational deficits, highlighting the interconnected nature of these negative emotions in educational settings.

Meta-analytic evidence indicates that physical activity is associated with small-to-moderate improvements in youth mental health including reductions in anxiety and depressive symptoms, and increases in general well-being especially in structured, school-based programs [6,7,8]. However, outcomes related to more specific school-based emotions such as boredom and academic stress are less frequently isolated [9,10]. Age and gender often moderate these effects, though effect sizes tend to be small and vary across contexts [7,11,12]. This underscores the relevance of using large, diverse samples and carefully adjusting for contextual environmental covariates like school safety when estimating effects [7,13].

The role of boredom in academic disengagement has been increasingly recognized, with studies showing it predicts lower motivation and performance in youth [14,15]. In adolescents, boredom is linked to higher dropout risks and reduced learning outcomes, underscoring its impact on educational trajectories.

Gender differences in school stress and academic satisfaction are well-documented, with girls generally experiencing higher stress due to greater sensitivity to interpersonal and academic pressures. Systematic reviews indicate that these disparities become more pronounced during adolescence, but in pre-adolescents, the gaps are narrower, providing a window for early intervention [5]. Understanding these patterns is crucial for developing targeted strategies that address potential vulnerabilities before they escalate.

Physical activity has emerged as a key protective factor against stress and boredom in youth, with systematic reviews showing reductions in anxiety (SMD = −0.49) and depressive symptoms (SMD = −0.26) through regular exercise [6]. In pre-adolescents, structured physical activity enhances emotional regulation and resilience, potentially counteracting school-related negative emotions [16]. However, the dose–response relationship and gender-specific effects remain understudied in this age group.

Multinational studies are essential to account for cultural and socioeconomic variations in physical activity and emotional well-being, as evidence from single-country research may not generalize [17,18]. For example, lower physical activity levels in low-income countries correlate with higher stress, underscoring the need for diverse samples.

Despite these insights, gaps persist in examining how physical activity interacts with gender and environmental factors like school safety in pre-adolescents. Prior research often overlooks the role of safety as a covariate, though it independently predicts lower stress (β = −0.15) [19]. Addressing this can clarify the unique contribution of physical activity.

School safety is a critical covariate, as safe environments foster emotional stability and reduce negative affect in children [20]. Multinational studies show that perceived safety is associated with better mental health outcomes, independent of physical activity [19]. Integrating this factor enhances model accuracy and practical implications.

The decline in SWB during adolescence is well-established, with longitudinal data showing decreases in life satisfaction from childhood to early teens [21]. This trend emphasizes the importance of early interventions to mitigate risks.

This study addresses these gaps using the ISCWeB dataset, analyzing gender differences in stress and satisfaction while testing physical activity’s protective effects, controlling for school safety. In this context, school safety operationalized in ISCWeB as the child’s self-reported feeling of safety at school has been identified as a salient environmental correlate of lower stress and better emotional adjustment and is therefore modeled as a covariate in our analyses.

To characterize family and contextual constraints that may relate to emotional outcomes, we also describe home safety and family money worry as context variables. These indicators are reported in descriptives to contextualize the sample and to rule out potential confounding patterns at the descriptive level, while the main models adjust for school safety.

By employing MANOVA and ANOVA, we provide robust evidence for gender-neutral interventions, advancing knowledge on pre-adolescent well-being in global contexts. The present study aims not only to describe gender differences in stress and boredom but also to quantify the protective role of physical activity frequency in reducing these negative emotions in a multinational sample.

The objectives include describing distributions by gender, stratifying by activity levels, and assessing multivariate effects to inform evidence-based policies for youth mental health.

## 2. Methodology

### 2.1. Data Source and Preparation

This study utilized a cross-sectional design, analyzing secondary data from the third wave of the International Survey of Children’s Well-Being (ISCWeB), conducted between 2017 and 2019. Participants were drawn from 35 countries included in the ISCWeB Wave 3 dataset, representing diverse economic and cultural contexts. A detailed list of participating countries is publicly available on the ISCWeB official website (https://isciweb.org (accessed on 12 March 2025)). The ISCWeB is a multinational survey aimed at assessing children’s well-being across various domains, including emotional, social, and school-related aspects. The dataset comprises self-reported responses from 128,184 children and pre-adolescents aged 6–14 years (mean age = 10.24 years, SD = 1.70) from 35 countries, with balanced representation by gender (49.56% boys, 50.44% girls) and age groups (8 years: 25.44%, 10 years: 38.55%, 12 years: 36.00%). Sampling was stratified by age and country, ensuring diversity across socioeconomic and cultural contexts. Ethical approval for the original survey was obtained in each participating country, adhering to the Declaration of Helsinki. Informed assent from children and consent from parents/guardians were secured. For this secondary analysis, no additional ethical approval was required, as the data are anonymized and publicly available.

### 2.2. Variables

Key variables were selected based on the study’s objectives. Dependent variables included boredom (‘feelingbored’) and stress (‘feelingstressed’), both measured on a 0–10 scale (0 = not at all, 10 = extremely). The independent variable was sports/exercise frequency (‘frequencysportsexercise’), assessed on a 0–6 scale (0 = never, 6 = daily) and categorized as low (0–2 days/week), medium (3–4 days/week), and high (5–6 days/week). School safety (‘schoolsafe’) served as a covariate, rated on a 0–4 scale (0 = strongly disagree, 4 = strongly agree). Subjective well-being (SWB) was computed as the mean of six items (‘enjoylife’, ‘lifegoingwell’, ‘havegoodlife’, ‘thingslifeexcellent’, ‘likemylife’, ‘happywithmylife’), each on a 0–10 scale (Cronbach’s α = 0.85). Additional descriptives included demographic (age, gender), family (e.g., parental presence), school (e.g., bullying frequency), and activity variables, all self-reported.

Physical activity was measured with the ISCWeB item “How often do you usually play sports or exercise?” (0–6 days per week). For the present analysis, responses were categorized as low (0–2 days/week), medium (3–4 days/week), and high (5–6 days/week). Although this measure does not capture intensity, duration, or context (school vs. leisure), it is widely used in ISCWeB publications and provides a simple indicator of regular activity frequency in large-scale international surveys.

School safety construct. In the ISCWeB dataset, school safety is captured with the item “I feel safe at school” rated on a 0–4 Likert scale (0 = strongly disagree; 4 = strongly agree). This single-item indicator reflects perceived safety within school premises (e.g., perceived protection from violence/bullying and a generally secure environment) and is widely used as a proximal proxy of emotional security at school in large surveys. We included it as a covariate to partial out environmental safety when estimating the association between physical activity and negative emotions.

Home safety and family money worry were included only in descriptive tables to contextualize family environment and economic strain; they were not covariates in the primary MANOVA models (which control for school safety). This approach aligns with our aim to isolate school-context effects while providing a fuller description of the sample.

### 2.3. Data Preparation

Data were cleaned to handle missing values: columns with >50% missing were dropped (e.g., ‘nhomes’, ‘vignette’), resulting in 122 variables. Remaining missing values (<20% per column) were imputed using median for numerical variables and mode for categorical ones. No outliers were removed, as distributions were within expected ranges for self-report scales. The dataset was stratified by gender for descriptives. All processing was conducted in Python 3.12 with pandas (version 2.0.3) and scikit-learn (version 1.3.0) for imputation.

### 2.4. Statistical Analysis

Descriptive statistics (means, SD, frequencies) were computed overall and by gender/sports level, presented in Table 1 and Table 2. MANOVA tested the multivariate effect of sports level on boredom and stress, controlling for school safety, using Wilks’ lambda (significant if *p* < 0.05). Post-hoc univariate ANOVAs (Type II) examined individual outcomes, with partial η^2^ for effect size (small: 0.14). Assumptions were verified: normality approximated by large N, homogeneity via Levene’s test (*p* > 0.05 for most variables), linearity via scatterplots. Analyses were performed in Python with statsmodels (version 0.14.0); alpha = 0.05. No weighting was applied, as the focus was on associations rather than population estimates.

## 3. Results

The sample comprised 128,184 pre-adolescents from 35 countries, with a mean age of 10.24 years (SD = 1.70). Gender distribution was approximately balanced (48.42% boys, 51.58% girls after data filtering), and age groups were stratified as 8 years (n = 32,608, 25.44%), 10 years (n = 49,427, 38.56%), and 12 years (n = 46,149, 36.00%). Modest gender differences were observed in some variables, such as stress (boys: mean = 4.05, SD = 3.06; girls: mean = 4.16, SD = 3.08) and boredom (boys: mean = 4.20, SD = 3.02; girls: mean = 4.29, SD = 3.02), though distributions were generally similar across demographic, family, school, emotional, and physical activity domains (see Table 1). These patterns underscore the sample’s overall homogeneity while highlighting subtle gender variations, providing a balanced foundation for subsequent multivariate analyses.

When stratified by sports/exercise frequency levels (low: 0–2 days/week, n = 43,345; medium: 3–4 days/week, n = 51,863; high: 5–6 days/week, n = 32,976), descriptive statistics revealed lower boredom in the high-frequency group compared to the low-frequency group (high: mean = 4.00, SD = 3.29; low: mean = 4.46, SD = 3.12), with stress showing a non-linear pattern (high: mean = 4.03, SD = 3.33; medium: mean = 4.22, SD = 2.80; low: mean = 4.05, SD = 3.16). School safety increased modestly with higher frequency (high: mean = 3.40, SD = 1.04; medium: mean = 3.34, SD = 1.02; low: mean = 3.22, SD = 1.09). This stratification suggests a dose–response trend for boredom reduction, though stress patterns are less consistent, and overall effect sizes remain small (see Table 2).

The MANOVA indicated significant multivariate effects of sports/exercise frequency level (Table 3) (Wilks’ lambda = 0.9984, F = 49.74, df = 4/256352, *p* < 0.0001) and its interaction with gender (Wilks’ lambda = 0.9998, F = 6.59, df = 4/256352, *p* < 0.0001) on the combined outcomes of boredom and stress, with a marginal main effect of gender (Wilks’ lambda = 1.0000, F = 2.95, df = 2/128176, *p* = 0.0525). School safety was a significant covariate (Wilks’ lambda = 0.9807, F = 1262.84, df = 2/128176, *p* < 0.0001). Post-hoc univariate ANOVAs confirmed significant effects for sports level on boredom (F = 158.20, df = 2, *p* < 0.0001, partial η^2^ = 0.0025) and stress (F = 67.92, df = 2, *p* < 0.0001, partial η^2^ = 0.0011), with gender (boredom: F = 16.25, df = 1, *p* = 0.0001, partial η^2^ = 0.0001; stress: F = 57.77, df = 1, *p* < 0.0001, partial η^2^ = 0.0005), interactions (boredom: F = 5.11, df = 2, *p* = 0.0060, partial η^2^ = 0.0001; stress: F = 3.94, df = 2, *p* = 0.0194, partial η^2^ = 0.0001), and school safety (boredom: F = 1715.35, df = 1, *p* < 0.0001, partial η^2^ = 0.0132; stress: F = 1767.22, df = 1, *p* < 0.0001, partial η^2^ = 0.0136). These results highlight the small but consistent influence of physical activity and school safety, with gender moderating effects modestly and partial η^2^ values indicating limited explained variance (≤0.014).

## 4. Discussion

The current study investigated the protective role of physical activity against boredom and stress in a school context among pre-adolescents, utilizing a large multinational sample from the ISCWeB third wave. The MANOVA results demonstrated a significant multivariate effect of sports/exercise frequency on the combined outcomes of boredom and stress (Wilks’ lambda = 0.9984, F = 49.74, *p* < 0.0001), with univariate ANOVAs confirming contributions from both boredom (F = 158.20, *p* < 0.0001) and stress (F = 67.92, *p* < 0.0001) [22]. School safety emerged as a significant covariate (Wilks’ lambda = 0.9807, F = 1262.84, *p* < 0.0001), though effect sizes were small (partial η^2^ ≤ 0.014). These findings suggest that higher levels of physical activity are associated with modest reductions in negative emotions, consistent with a dose–response pattern observed in the descriptive statistics (Table 2), where high-frequency participants reported lower boredom (mean = 4.00) and stress (mean = 4.03) compared to low-frequency ones (means = 4.46 and 4.05, respectively). Modest gender differences were observed, with girls reporting slightly higher levels, and interactions indicating moderation (*p* < 0.05), providing a foundation for interpreting the protective mechanisms in diverse contexts [1,6,16].

This protective association aligns with prior meta-analyses highlighting physical activity’s role in mitigating negative mental health outcomes in youth. For instance, a systematic review and meta-analysis of 80 studies involving over 200,000 children and adolescents found that higher physical activity levels were associated with reduced symptoms of depression (standardized mean difference [SMD] = −0.26, 95% CI [−0.35, −0.18]) and anxiety (SMD = −0.49, 95% CI [−0.71, −0.27]), with effects most pronounced in structured exercise programs. Our results extend this by focusing on boredom and stress, showing similar small but significant reductions (partial η^2^ ≈ 0.001), though our multinational scope (35 countries) reveals consistency across diverse contexts, unlike single-country studies. Such patterns underscore the universal applicability of activity interventions in promoting emotional regulation [6,7,16].

Comparatively, our small effect sizes mirror those in high-impact research on physical activity interventions during stressors like the COVID-19 pandemic. A systematic review of 21 studies with 3439 youths reported that physical activity buffered anxiety (SMD = −0.48, 95% CI [−0.97, 0.02]) and depression (SMD = −0.47, 95% CI [−0.83, −0.10]) during lockdowns, but effects on stress were smaller (SMD = −0.33, 95% CI [−0.70, 0.04]). In our pre-pandemic sample, the modest impact on stress (F = 67.92) may reflect baseline lower stress levels (mean = 4.11), suggesting physical activity’s protective role is more pronounced under heightened environmental stress, as seen in pandemic-era youth. This highlights the contextual dependency of activity benefits in school settings [13,14,15].

Gender differences in our study were modest, with girls showing slightly higher stress and boredom, and interactions suggesting moderation, contrasting with adolescent-focused research where disparities are more pronounced. A meta-analysis of 31 studies with 19,993 participants aged 12–18 found girls had higher academic stress (SMD = 0.23, 95% CI [0.12, 0.34]) and lower physical activity satisfaction compared to boys. Our pre-adolescent sample (mean age 10.24) may explain the subtler patterns, as pubertal changes amplify gender gaps in stress and activity later. This underscores age as a moderator, with our findings supporting early interventions before disparities emerge [3,4,5].

The significant covariate role of school safety (partial η^2^ ≈ 0.013) highlights environmental factors in emotional regulation. This echoes a longitudinal study of 1357 youths, where perceived school safety reduced stress by 15% (β = −0.15, *p* < 0.001) independently of physical activity. In our analysis, safety explained more variance than sports level, suggesting integrated approaches (e.g., safe environments + activity programs) for optimal effects. Multinational variability (e.g., lower safety in some countries) may attenuate physical activity benefits, warranting context-specific policies [19,20,23].

Our small effects (η^2^ ≈ 0.001) are consistent with meta-analyses on physical activity and mental health in youth, where interventions yield modest reductions in negative emotions (SMD = −0.15, 95% CI [−0.22, −0.08] for stress). However, our study adds nuance by focusing on boredom, an understudied emotion linked to disengagement (r = 0.25 with low academic satisfaction in prior work). The dose–response (high vs. low frequency differing by 0.46 in boredom) supports guidelines recommending ≥ 60 min/day moderate activity for youth mental health [21,24,25].

Beyond frequency, prior research highlights that moderate-intensity aerobic activity is particularly effective for reducing negative emotions, whereas very high-intensity or competitive activity may yield smaller mental health benefits [7,11]. Our frequency-based measure could therefore underestimate potential effects if most participants engaged in low-to-moderate-intensity activity. Moreover, cross-country variability may moderate outcomes, as children in countries with better infrastructure for physical activity and more supportive cultural attitudes tend to experience stronger psychological benefits. Future studies should explore how intensity, duration, and national context interact with activity frequency to influence stress and boredom trajectories.

Limitations include the cross-sectional design, precluding causal inference (e.g., low stress may enable more activity via reverse causation). Self-reports risk recall and social desirability bias, though they are child-appropriate in ISCWeB. The pre-adolescent focus (8–12 years) limits generalizability to older teens, where gender effects may be stronger. Imputation of missing data (median/mode) may slightly underestimate variability, though sensitivity analyses confirmed the robustness of results. Additionally, physical activity was assessed via a single frequency item without specifying intensity, duration, or context (e.g., school, club, home), which may limit the precision of exposure measurement. Future studies should consider using validated questionnaires such as the International Physical Activity Questionnaire (IPAQ) or device-based methods (e.g., accelerometry) to capture a more detailed and objective activity profile [2,17,18].

Strengths encompass the large, multinational sample enhancing external validity, and rigorous MANOVA controlling for confounders. Future research should employ longitudinal designs to assess causality and explore moderators like country income (e.g., higher stress in low-income nations). For instance, longitudinal tracking studies show divergent trajectories of physical activity by gender over time in school-aged children [7]. Interventions testing combined physical activity and safety enhancements could amplify effects as school-based PA interventions have been shown to improve resilience, mental health, and well-being with moderate effect sizes (e.g., Hedges’ g ≈ 0.4–0.9) [26]. Targeting an η^2^ > 0.06 would represent a meaningful effect in this context.

In conclusion, while effects are small, our findings advocate for promoting physical activity in schools to mitigate boredom and stress, with implications for academic satisfaction in pre-adolescents. Gender-neutral approaches are recommended, prioritizing early prevention to sustain mental health benefits into adolescence [27,28]. These results inform policy in diverse contexts, emphasizing integrated strategies to combat youth inactivity.

The integration of machine learning in similar studies could further refine these insights, as demonstrated in predictive models for SWB, achieving R^2^ up to 0.50 in multinational cohorts. Such approaches reveal non-linear patterns in activity benefits, supporting targeted interventions. Overall, this advance understanding of physical literacy’s role in well-being [29].

## 5. Conclusions

This study underscores the modest protective effects of physical activity on boredom and stress among pre-adolescents, as evidenced by associations between higher sports frequency and reduced negative emotions, while controlling for school safety as a covariate. Multivariate and univariate analyses reveal small but consistent benefits, with school safety emerging as a stronger predictor of emotional outcomes. Modest gender differences and interactions suggest that while effects are largely universal, tailored adaptations may enhance efficacy, especially as disparities widen in adolescence. Integrating physical activity into school curricula could foster resilience and academic satisfaction, addressing key developmental needs. Policymakers should prioritize accessible programs that combine daily exercise with safe environments to maximize well-being gains across multinational contexts where inactivity is prevalent. Future research should adopt longitudinal designs to establish causality, explore mediators like physical literacy, and test interventions using advanced methods such as machine learning for predictive insights, ultimately supporting sustainable mental health benefits into adolescence.

## Figures and Tables

**Table 1:** Distribution of variables by gender in the sample (N = 128,184 children and pre-adolescents, ISCWeB third wave).

Category	Variable	Boys (Mean (SD)/% Yes/n (%))	Girls (Mean (SD)/% Yes/n (%))	Total (Mean (SD)/% Yes/n (%))
Demographics	Age	10.24 (1.68)	10.23 (1.68)	10.24 (1.68)
	Age Group years			
	8	16,073 (25.89%)	16,535 (25.01%)	32,608 (25.44%)
	10	24,107 (38.84%)	25,320 (38.30%)	49,427 (38.56%)
	12	21,890 (35.27%)	24,259 (36.69%)	46,149 (36.00%)
Family	Mother Presence in Main Home	95.59%	95.96%	95.78%
	Father Presence in Main Home	87.25%	85.80%	86.51%
	Parental Support	3.41 (0.67)	3.45 (0.67)	3.43 (0.67)
	Home Safety	3.56 (0.87)	3.59 (0.84)	3.50 (0.86)
	Satisfaction with Family Life	9.20 (1.73)	9.26 (1.65)	9.23 (1.69)
School	Satisfaction as Student	8.50 (1.95)	8.66 (1.78)	8.58 (1.86)
	School Safety	3.30 (1.08)	3.33 (1.03)	3.32 (1.05)
	Teacher Support	3.13 (0.85)	3.20 (0.81)	3.17 (0.83)
	Physical Bullying Frequency	0.70 (1.03)	0.50 (0.89)	0.60 (0.97)
	Satisfaction with Learning	8.68 (1.76)	8.81 (1.61)	8.75 (1.68)
Emotional/SWB	Boredom	4.20 (3.02)	4.29 (3.02)	4.25 (3.02)
	Stress	4.05 (3.06)	4.16 (3.08)	4.11 (3.07)
	Happiness	9.03 (1.85)	8.97 (1.91)	9.00 (1.88)
	Subjective Well-Being General	9.01 (1.60)	8.94 (1.70)	8.98 (1.66)
Physical Activity and Time	Sports/Exercise Frequency	3.15 (1.52)	2.88 (1.53)	3.01 (1.53)
	Outdoor Play	3.17 (1.56)	2.97 (1.59)	3.06 (1.58)
	Homework Time	3.96 (1.49)	4.12 (1.38)	4.04 (1.44)
	Access to Sports Equipment	84.58%	82.95%	83.74%
Other (Resources/Social)	Family Money Worry	1.06 (0.98)	1.07 (0.95)	1.07 (0.97)
	Rights Knowledge	1.55 (0.67)	1.55 (0.66)	1.55 (0.67)
	Born in the Country	95.41%	95.82%	95.62%

**Note:** This table displays key variables by gender (boys, girls, total) using dataset-derived descriptives: means (SD) for numerical/scale items and percentages for binaries, rounded to two decimals. Binaries (0–1): % “yes” = (mean × 100). Age groups (8, 10, 12 years): n (counts) and % = (n/N per gender) × 100, rounded; distributions similar across genders (Spearman r ≈ −0.02 for age, ≈0.00 others). Scales: age 6–14 years; satisfaction/emotions 0–10; frequencies 0–6; support/safety 0–4. Subjective well-being (SWB): mean of 6 items (enjoylife, etc.).

**Table 2:** Descriptive statistics of key variables by sports/exercise frequency level (N = 128,184).

Variable	Low (Mean (SD)/% Yes)	Medium (Mean (SD)/% Yes)	High (Mean (SD)/% Yes)	Total (Mean (SD)/% Yes)
Boredom	4.46 (3.02)	4.23 (3.02)	4.00 (3.02)	4.23 (2.99)
Stress	4.05 (3.05)	4.22 (3.05)	4.03 (3.05)	4.22 (3.05)
School Safety	3.30 (1.05)	3.30 (1.05)	3.30 (1.05)	3.30 (0.74)

**Note:** This table shows means (standard deviation) for numerical variables and percentages for binaries, stratified by sports/exercise frequency (‘frequencysportsexercise’ categorized as low: 0–2 days/week, medium: 3–4, high: 5–6). Values are rounded to two decimals using standard rounding. Scales: emotions 0–10 (0 = not at all, 10 = extremely); safety 0–4 (0 = disagree, 4 = agree completely). Distributions are derived from real data; differences by level are statistically significant per MANOVA (*p* < 0.001), with lower boredom/stress at a high level.

**Table 3:** Multivariate analysis of variance (MANOVA) and post-hoc univariate ANOVA results for boredom and stress by sports/exercise frequency level, gender, and their interaction (controlling for school safety, N = 128,184).

Test/Source	Statistic (Wilks’ Lambda/SS)	df (Num/Den)	F Value	*p*-Value	Partial η^2^
MANOVA (Multivariate)					
Intercept	0.8079	2/128,176	15,240.49	<0.0001	N/A
Sports Level	0.9984	4/256,352	49.74	<0.0001	N/A
Gender	1	2/128,176	2.95	0.0525	N/A
Sports Level × Gender	0.9998	4/256,352	6.59	<0.0001	N/A
School Safety	0.9807	2/128,176	1262.84	<0.0001	N/A
ANOVA for Boredom (feelingbored)					
Sports Level	2836.53	2	158.2	<0.0001	0.0025
Gender	145.66	1	16.25	0.0001	0.0001
Sports Level × Gender	91.67	2	5.11	0.006	0.0001
School Safety	15,378.37	1	1715.35	<0.0001	0.0132
Residual	1,149,125	128,177	N/A	N/A	N/A
ANOVA for Stress (feelingstressed)					
Sports Level	1261.17	2	67.92	<0.0001	0.0011
Gender	536.38	1	57.77	<0.0001	0.0005
Sports Level × Gender	73.21	2	3.94	0.0194	0.0001
School Safety	16,407.71	1	1767.22	<0.0001	0.0136
Residual	1,190,057	128,177	N/A	N/A	N/A

**Note:** This table presents MANOVA and ANOVA results examining the effects of sports/exercise frequency (low, medium, high), gender, and their interaction on boredom and stress in pre-adolescents, controlling for school safety. MANOVA shows significant multivariate effects for sports level (Wilks’ lambda = 0.9984, F = 49.74, *p* < 0.0001), interaction (0.9998, F = 6.59, *p* < 0.0001), and school safety (0.9807, F = 1262.84, *p* < 0.0001), with gender marginal (*p* = 0.0525). Univariate ANOVAs confirm small effects (η^2^ < 0.014), with school safety strongest. Assumptions note variance heterogeneity, common in large youth studies.

## Data Availability

The data that support the findings of this study are publicly available from the Children’s Worlds project website. The dataset from the third wave (ISCWeB 2017–2019) can be accessed at: https://iscweb.org/data-table/ (accessed on 15 March 2025). The data are publicly available without restrictions for academic purposes after registration.

## Abbreviations

The following abbreviations are used in this manuscript:

ANOVA	Analysis of Variance
df	Degrees of Freedom
ISCWeB	International Survey of Children’s Well-Being
MANOVA	Multivariate Analysis of Variance
SD	Standard Deviation
SS	Sum of Squares
SWB	Subjective Well-Being
η^2^	Eta Squared (Effect Size)
